# Metabolic Switching of Tumor Cells under Hypoxic Conditions in a Tumor-on-a-chip Model

**DOI:** 10.3390/mi11040382

**Published:** 2020-04-04

**Authors:** Valentina Palacio-Castañeda, Lucas Kooijman, Bastien Venzac, Wouter P.R. Verdurmen, Séverine Le Gac

**Affiliations:** 1 Department of Biochemistry, Radboud Institute for Molecular Life Sciences (RIMLS), Radboud University Medical Center, Geert Grooteplein 28, 6525 GA Nijmegen, The Netherlands; Valentina.Palacio-Castaneda@radboudumc.nl; 2 Applied Microfluidics for BioEngineering Research, TechMed Center & MESA+ Institute for Nanotechnology, University of Twente, Postbus 217, 7500AE Enschede, The Netherlands; l.j.kooijman@utwente.nl (L.K.); b.venzac@utwente.nl (B.V.)

**Keywords:** Tumor-on-a-chip, Hypoxia, pH, Microfluidics, PDMS, Cell metabolism

## Abstract

Hypoxia switches the metabolism of tumor cells and induces drug resistance. Currently, no therapeutic exists that effectively and specifically targets hypoxic cells in tumors. Development of such therapeutics critically depends on the availability of in vitro models that accurately recapitulate hypoxia as found in the tumor microenvironment. Here, we report on the design and validation of an easy-to-fabricate tumor-on-a-chip microfluidic platform that robustly emulates the hypoxic tumor microenvironment. The tumor-on-a-chip model consists of a central chamber for 3D tumor cell culture and two side channels for medium perfusion. The microfluidic device is fabricated from polydimethylsiloxane (PDMS), and oxygen diffusion in the device is blocked by an embedded sheet of polymethyl methacrylate (PMMA). Hypoxia was confirmed using oxygen-sensitive probes and the effect on the 3D tumor cell culture investigated by a pH-sensitive dual-labeled fluorescent dextran and a fluorescently labeled glucose analogue. In contrast to control devices without PMMA, PMMA-containing devices gave rise to decreases in oxygen and pH levels as well as an increased consumption of glucose after two days of culture, indicating a rapid metabolic switch of the tumor cells under hypoxic conditions towards increased glycolysis. This platform will open new avenues for testing anti-cancer therapies targeting hypoxic areas.

## 1. Introduction

Oxygen has a primordial role in biological systems and tissues. Distinct tissues in the human body are exposed to different oxygen pressures, ranging from atmospheric conditions in the air-exposed regions of lung tissues (21% O_2_) to below 5% O_2_ in, for instance, the female reproductive track. In disease, and in particular in cancerous tissues, variations in oxygen levels are widespread due to the presence of an irregular vasculature, giving rise to regions that are normoxic, hypoxic or even almost fully anoxic [1,2]. Specifically, gradients of oxygen tension are found from the blood vessels to areas further apart [3]. Cells in turn adapt their metabolism to these variations in oxygen availability, with an increased glycolytic behavior [4] and significant accumulation of waste products in regions far away from the blood vessels. Besides adapting their metabolism, cells exhibit additional altered phenotypic characteristics in these remote areas: they do not proliferate as much as their counterparts in the vicinity of the blood vessels; they become quiescent or even apoptotic or necrotic [5]; and they express a different repertoire of membrane proteins [6]. More importantly, this shift in phenotypes is accompanied by a change in the cell response to chemotherapeutic [7], radiotherapeutic [8] and as more recently observed, immunotherapeutic [9] treatments. Furthermore, there is a notable increase in their aggressiveness, and metastatic potential [10]. Altogether, to accurately emulate the in vivo situation and to also account for this variety in cell phenotypes, it is essential to control and incorporate oxygen tension differences when designing in vitro tumor models.

Microfluidic technology has become a game-changer to conduct in vitro experiments on cells. In contrast to conventional formats, microfluidics provides exquisite control on any physical and chemical parameter of the cell culture in the device at the micrometer scale, due to the reduction in device dimensions, the larger surface-to-volume ratio, and the laminar character of the flow [11,12]. Similarly, these miniaturized devices lend themselves well to the creation of stable gradients of soluble factors [13], or other physical and chemical parameters (temperature, gas tension) [14]. Finally, in microfluidic devices, cells are not only grown as monolayers, but also as 3D constructs [15], cell-laden hydrogel materials [16,17,18], as differentiated epithelium on porous membranes [19,20,21], as co-culture systems [22], or even as complex models aiming at emulating the architecture and/or function of an organ [23]. The most widely used material to fabricate microfluidic devices is PDMS or polydimethylsiloxane [24,25]. Its popularity is explained by the ease of device fabrication, without any requirement for a dedicated cleanroom environment and with minimal training, its relatively low price, its elastomeric properties, and its gas permeability. However, while the latter property is of great interest to ensure proper transport of oxygen and carbon dioxide to and from cells grown in the device, it impedes the ability to regulate oxygen concentrations and create a hypoxic microenvironment in 3D culture models.

A variety of platforms have been reported to address this significant limitation of PDMS and to apply well-defined oxygen tension or to create oxygen gradients across cell cultures, as recently reviewed [26,27]. These platforms broadly follow a few working principles: (i) the gas exchange through the chip material is blocked or limited; (ii) oxygen is delivered or eliminated from a fluid source; and (iii) oxygen gradients develop from an oxygen source to the core of the tissue model due to the cell consumption. One first and straightforward approach consists of fabricating the devices from gas-impermeable materials such as glass [28], SU-8 [29] or polystyrene [16]. Alternatively, polymer films with low oxygen permeability were embedded in PDMS devices to induce hypoxic conditions on 2D [30] or 3D cell cultures [31]. In a different approach, and by taking advantage of the gas permeability of PDMS or similar silicone materials, dedicated channels perfused with pre-conditioned fluids were placed on the top [15,32,33,34] or next to culture chambers [35,36], allowing a quick switch of the oxygen tension in a 3D tumor model [37], to create oxygen gradients in cellular models [38,39] or to simply supply enough oxygen to hepatocytes [15]. Along a similar line, solutions supplemented with an oxygen-scavenging chemical were utilized to create hypoxic conditions [40] or oxygen gradients [41,42,43,44,45], in combination in one report with a thick oxygen-impermeable material (PMMA or polymethylmethacrylate) sheet placed on the top of the device [46].

In this work, we report a tumor-on-a-chip platform in which normoxic or hypoxic conditions, as well as gradients of oxygen can reproducibly be generated in a cell-laden hydrogel. Importantly, these microfluidic devices were fabricated without a cleanroom and operated in a standard incubator. The fluidic structures were fabricated from PDMS and bonded to glass. To block oxygen diffusion through the PDMS from the top of the device, a 175-μm thick sheet of PMMA was introduced in the PDMS roof of the device at a controlled height, before PDMS curing. We first characterized the device with oxygen-sensitive probes to confirm the PMMA sheet could efficiently limit oxygen diffusion in the culture chamber and demonstrated the possibility to create hypoxic conditions. Next, a 3D tumor model was created in the middle of the device, by confining first a 2-mm wide cell-laden hydrogel containing U-251 MG glioblastoma cells between two cotton threads. These barriers were then pulled out and removed from the device, to create a cell-laden hydrogel slab flanked by two perfusion channels. Incorporation of the PMMA sheet led to reductions in oxygen tension in the culture without affecting the viability of the U-251 MG cells. Facing hypoxic conditions, the cells switched metabolically towards a more glycolytic behavior as reflected by increased glucose uptake and acidity in the hydrogel.

## 2. Materials and Methods

### 2.1. Fabrication of the Microfluidic Systems

**Figure 1:**
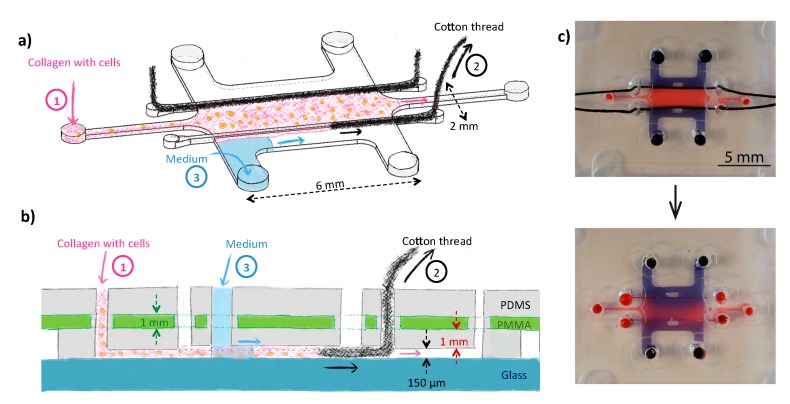
Schematic representation of the tumor-on-a-chip device (top (**a**) and side (**b**) views) consisting of a large cell culture chamber in which collagen supplemented with tumor cells is introduced. To confine the cell-laden hydrogel in the center of the device, removable filaments (cotton threads, here) were first introduced in the device, and pulled out of the device before gelation of the collagen (a & b). Culture medium with 21% O_2_ was added in the two side channels during the experiments. The device was fabricated from PDMS and bonded to a glass substrate; the PDMS layer comprised a 175-μm thick PMMA film to prevent O_2_ from freely diffusing into the cell culture chamber so as to be able to establish gradients of O_2_ in the tumor-on-a-chip model between the perfusion channels and the center of the 3D cellular model. (**c**) Pictures of the fabricated device loaded with dyes (blue: Trypan blue; red: dextran labeled with fluorescein and TAMRA) before (top) or 5 min after (bottom) removing the cotton threads. On these pictures, all reservoirs have a 1.5-mm diameter.

The microfluidic device consists of a 6-mm long, 2-mm wide and 150-µm high culture chamber connected to two 380-µm wide, 4-mm long inlet/outlet channels ending in two inlet and outlet reservoirs (diameter 1.5 mm). The culture chamber is flanked by two medium channels (6-mm long, 1-mm wide and 150-µm high), each with two 1.5-mm diameter reservoirs. Two guiding channels (8-mm long, 187.5-μm wide and 200-µm high) are located between the culture chamber and the perfusion channels for the insertion of two removable filaments, as depicted in Figure 1a, with 1.5-mm diameter inlets.

Molds for soft lithography were produced in-house using a digital light processing 3D printer (Hunter, FlashForge, Zhejiang, China) and a photosensitive resin (Deep Black resin, Fun-To-Do, Alkmaar, The Netherlands). The molds (Figure S2) were printed horizontally on a 2-mm thick base, surrounded by 1-mm high support structures and 3-mm high walls forming a reservoir. The *z*-resolution was 50 µm and the lateral resolution 62.5 µm, which corresponds to the pixel size of the projected images. The exposure time was set at 20 s for the first 8 layers and 2 s for the rest of the layers. After printing, the structure was quickly rinsed with acetone and ethanol, dried with air and exposed to an in-house optimized post-treatment to eliminate the curing inhibition of polydimethylsiloxane (PDMS) in contact with the 3D-printed molds [47]: the molds were first UV cured under 405 nm LEDs during 15 min at 14 mW/cm^2^ in a home-made UV oven, then placed for 2 h at 120 °C on a hotplate, while being covered by a glass dish.

A 10:1 (w/w) PDMS mixture of base and curing agent (Sylgard 184 Dow Corning, Midland, MI, USA) was poured on the mold after thorough degassing in vacuum. A 175-µm thick PMMA film (Goodfellow, Huntingdon, England) was punched with a 3-mm diameter puncher and placed in the PDMS mixture on the 1-mm thick support structure of the mold. During this process, care was taken to align the punched holes in the PMMA manually with the ten reservoir structures on the 3D printed structures. The PDMS mixture was added next to fill the 2-mm high reservoir. After 3 h of curing in an oven at 60 °C, the device was released from the mold, inlets were punched through the PDMS and pre-formed holes on the PMMA sheet with 1 mm and 0.5 mm punchers. Two mercerized cotton threads (reference col. 5001, Gütermann, Gutach im Breisgau, Germany) with a linear density of 33 Tex or g/km (corresponding to an approximate diameter of 250 µm without tension) were carefully placed in the guiding channels and through the dedicated inlets, and maintained in place by firmly pulling them through both inlets. The PDMS/PMMA/cotton assembly was finally bonded to 1-mm thick glass slides after air plasma treatment (Cute, Femto Science, South Korea). The entire fabrication process is provided as supplementary information (Figure S1).

### 2.2. Characterization of the Microfluidic Systems under Normoxic and Hypoxic Conditions

To characterize the PMMA-PDMS devices, they were placed in a glove box (78 L, Aldrich^®^ AtmosBag, Sigma-Aldrich, Zwijndrecht, The Netherlands) connected to a N_2_ gas line to be able to create an anoxic environment surrounding the devices. For this characterization, a simplified version of the device was employed, consisting of a simple chamber (1-mm width; 6-mm length and 150-µm height), one inlet and one outlet channel (Figure S2), prepared in the same manner as the devices meant for cell culture, with a 175-µm thick PMMA sheet embedded inside the PDMS fluidic layer. As a control, devices were fabricated without this PMMA sheet to confirm the ability of the PMMA layer to block oxygen diffusion into the culture chamber. The oxygen level in the device was monitored optically using a solution of a commercially available oxygen-sensitive dye, tris(2,2′-bipyridyl)dichlororuthenium(II) hexahydrate (Ru(BPY)_3_) (Sigma-Aldrich) at 5 mg/mL. Specifically, the fluorescence intensity of these probes linearly correlates with the oxygen tension in the device, and the higher the intensity the lower the oxygen concentration [48]. This solution was placed in the glove box together with the microfluidic devices under anoxic conditions (N_2_) overnight to deplete oxygen, while protecting the dye from light. Next, two types of microfluidic devices were injected with the oxygen-sensitive solution, PDMS devices with a PMMA sheet and standard PDMS devices (no PMMA sheet), as a control. The inlets and outlets of the devices were carefully blocked with 2-mm stainless steel screws to ensure gas diffusion into the device would occur through their sides and not via the reservoirs. Devices were next transferred to a microscope in a sealed container to prevent any gas exchange with the environment before the start of the experiments. Devices were placed one at a time on the microscope stage under normal atmospheric conditions (21% O_2_, room temperature) and O_2_ diffusion into the devices was examined by monitoring the variations in the oxygen-sensitive probe fluorescence intensity in the entire culture chamber. Pictures were acquired at different intervals over a time period of more than 4 h (every 2 min in the first 10 min; every 5 min in the following 60 min; every 10 min in the following 60 min; and every 12 min in the last two hours), while limiting exposure of the dye to light (lamp on only when taking a picture; setup wrapped in aluminum foil; measurements conducted in a dark room). The dye was excited at a 450 nm and filtered for detection by a high-pass filter at 530 nm. Experiments were repeated on three different days on independent devices (three devices per condition). Images were analyzed using ImageJ software (NIH, Bethesda, MD, USA) in areas of 0.5 mm × 0.5 mm in the chamber, by determining the average fluorescence intensity value in this area. Data were analyzed as detailed in supplementary information (Figure S3), and as suggested in previous literature [42] using the Stern-Volmer relation and the relative fluorescence intensity values measured for 0% (start of the experiments) and 21% oxygen (end of the experiments for the PDMS only devices) in the device.

### 2.3. Cell Culture

The human glioblastoma astrocytoma U-251 MG cell line (kindly provided by Dr. Leenders, department of Biochemistry, Radboud University Medical Center) was cultured and maintained in high-glucose (4.5 g/L) Dulbecco’s Modified Eagle Medium (DMEM. Life Technologies, Thermo Fisher Scientific, Waltham, MA, USA) supplemented with 10% fetal calf serum (FCS) (PAN-Biotech, Aidenbach, Germany) and kept in a humidified incubator at 37 °C and 5% CO_2_. Cells were passaged when they reached 80% confluency using trypsin-EDTA (PAN-Biotech) for detachment.

### 2.4. Cell Culture in the Microfluidic Systems

Devices were sterilized before loading of the cell-laden hydrogel by submerging them for 20 min in a solution of 70% (v/v) ethanol. After this, the ethanol was removed by aspiration and the devices were placed in an oven to dry at 60 °C for 2 h. U-251 MG cells were detached and counted using a hemocytometer. The cell suspension was next centrifuged and resuspended in Hank’s Balanced Salt Solution (HBSS) at a concentration of 10^6^ cells/mL. The cells were stained with CellTrace Violet, CFSE (Invitrogen, Thermo Fisher Scientific) or CytoTell Red (AAT Bioquest, Sunnyvale, CA, USA) according to the manufacturer’s instructions. A solution of 4 mg/mL collagen (rat tail collagen type I, Corning, NY, USA) was prepared following the manufacturer’s instructions, with stained cells at a concentration of 40 × 10^6^ cells/mL. Immediately after mixing, the cell-laden hydrogel was loaded manually by pipetting into the device. Two minutes after loading, the cotton threads were removed, and devices placed in the incubator for 30 min to allow the collagen to polymerize. When the hydrogel was polymerized, medium containing 1% penicillin-streptomycin (Sigma-Aldrich), 2.5 μg/mL Amphotericin B (Sigma-Aldrich) and 0.5% gentamicin (Merck Millipore, Darmstadt, Germany) was added into the side channels and on top of the devices. The devices were then placed in a 145 mm × 20 mm petri dish with PBS in the bottom and kept in a humidified incubator at 37 °C and 5% CO_2_. The medium in the side channels and on top of the devices was refreshed after 24 h.

### 2.5. Validation and Characterization of Hypoxia in the Microdevices

U-251 MG cells were stained with the CytoTell Red dye prior to loading and embedded in a 4 mg/mL collagen solution. After 48 h of culture in the device, the medium in the side channels was replaced with a solution of 5 µM Image IT Hypoxia Green combined with propidium iodide (PI; Sigma-Aldrich) at a final concentration of 10 μg/mL. For pH characterization, the medium in the microdevice was exchanged with medium containing a dual-labeled pH indicator dextran (70,000 MW; labeled with fluorescein and tetramethylrhodamine (TAMRA) Sigma-Aldrich) at a concentration of 100 µg/mL. The microfluidic devices were incubated for 2 h at 37 °C before microscopy imaging. The ratio of fluorescence intensities of fluorescein and TAMRA was converted to pH values using a calibration curve based on the linear portion of the pH-dependency of the emission intensity of fluorescein in a diluted aqueous solution [49]. The TAMRA in the dual-labeled dextran is used as a reference dye with a pH-insensitive fluorescence quantum yield. For evaluation of the delivery and uptake of the fluorescent glucose analogue 2-deoxy-2-[(7-nitro-2,1,3-benzoxadiazol-4-yl)amino]-D-glucose (2-NBDG; Sigma-Aldrich), the medium in the side channels was replaced with a 200 µM 2-NBDG. The surface of the devices was also covered with the same solution and incubated for 90 min at 37 °C until microscopy imaging.

### 2.6. Confocal Microscopy Imaging

The microfluidic devices were imaged using a Leica TCS SP8 confocal microscope (Leica Microsystem SP8, Wetzlar, Germany). Image-IT Green Hypoxia Reagent was excited at 488 nm (detection: 496–550 nm), CellTrace Violet at 405 nm (detection: 413–460 nm), and CytoTell Red at 647 nm (detection: 656–750 nm). 2-NBDG was excited at 467 nm (detection: 475–575 nm), the fluorescein in the dual-labeled pH indicator dextran was excited at 488 nm (detection: 496–550 nm) and the TAMRA at 550 nm (detection: 558–620 nm). The objective HCX PL APO 10X numerical aperture 0.4 dry lens was used. For overview images, mosaic image acquisition was performed by recording pictures at 4–10 regions per device. The regions were subsequently merged using LASX software (Leica Microsystems). Image analysis was performed using Fiji software [50].

## 3. Results and Discussion

### 3.1. Design and Fabrication of the Microfluidic Systems

To generate a 3D tumor model in which oxygen gradients and hypoxic conditions can be induced, a microfluidic device was developed consisting of a 2 mm-wide central cell culture chamber, in which U-251 MG glioblastoma cells in a collagen matrix are added. This tumor tissue was flanked by two channels, in which standard culture medium containing 21% O_2_ was administered (Figure 1a). First, a liquid collagen-tumor cell suspension was injected between two cotton threads, acting as barriers to confine the hydrogel. The cotton threads were removed within ca. 2 min after loading of the collagen solution (Figure 1b). Figure 1c presents pictures of the actual device before and after removal of the cotton threads.

In previous works [16,17,18,29], rows of pillars were employed for the same purpose of confining a hydrogel matrix supplemented with cells in a microfluidic device to create a 3D cellular construct, and devices have notably been made in PDMS [17,18], polystyrene (PS) [16], cyclic olefin polymer (COP) [51], SU-8 [52] and poly(ethylene glycol) diacrylate (PEGDA) with a stereolithography 3D-printer [53]. A key element in that approach however is to accurately adjust the pillar geometry, their spacing and their surface properties (surface tension) to ensure proper confinement of the hydrogel in the dedicated chamber, as extensively discussed in previous articles [54,55]. The presence of pillars as largely impermeable barriers decreases the surface area of the interface between the hydrogel and the solution, and limits the choice of compatible hydrogels by setting requirements in terms of viscosity and surface tension. Therefore we decided to pursue an alternative approach that is furthermore also compatible with our 3D printer (4 k€, resolution 62.5 µm × 62.5 µm × 50 µm in x, y and z directions respectively). Noteworthy, most commercial digital light processing printing techniques do not offer the required resolution for the creation of pillars for hydrogel confinement, and only Kim et al. [53] successfully created such barriers, yet using a 10-k€ printer offering a higher resolution (27 µm × 27 µm × 10 µm) and an optimized custom resin, and high-viscosity hydrogels (PEGDA and agarose). At the same time, this limitation in resolution is counter-balanced by the low price, high prototyping speed (< 3 h) of the technique and the possibility to easily create structures with different heights in one step.

Building up on previously developed technology making use of removable filaments in a microfluidic device to create hydrogel structures [56,57] we first confined our 3D cell-hydrogel construct using Nylon filaments (VARIVAS Fluoro carbon Super tippet 2XN 0.235-mm diameter, Morris, Saitama, Japan) inserted in 200-µm high guiding channels through vertical inlets and outlets before bonding of the device. However, those large filaments were not deformable enough to bend and conform with the sharp angle existing at the inlets and outlets of the guiding channels, which significantly hampered bonding of the device with the glass slide, which was not the case for smaller height channels (80 µm) and thinner Nylon fibers (95-µm diameter) in the original paper introducing this technique. Therefore, we consecutively examined the applicability of more deformable filaments in the form of mercerized cotton threads. Due to their higher compliance, these cotton threads could adapt to the sharp angle between the guiding channel and the vertical inlets, ensuring good bonding of the chips. Next, collagen was successfully injected between the threads without leakage through these multi-filaments: the threads were indeed compressed inside the guiding channels, reducing the presence of inter-fiber spaces, and the commercial mercerization process (an alkaline treatment of the yarns in sodium hydroxide) increased the thread hydrophobicity. However, keeping the cotton threads in contact with the hydrogel for a few tens of minutes would still induce wicking and capillary pumping of the water-phase. Shortly after injection of the collagen (after ca. 2 min), threads were easily pulled out of the chip, following the guiding channel thanks to their lack of shape memory, leaving the partially gelled and more viscous collagen between the two guiding channels. Noteworthy, when using such cotton threads, the device should be thoroughly sterilized with ethanol before use, not to compromise cell viability during the experiments.

### 3.2. Generating Controlled Hypoxic Conditions in the Microfluidic System

**Figure 2:**
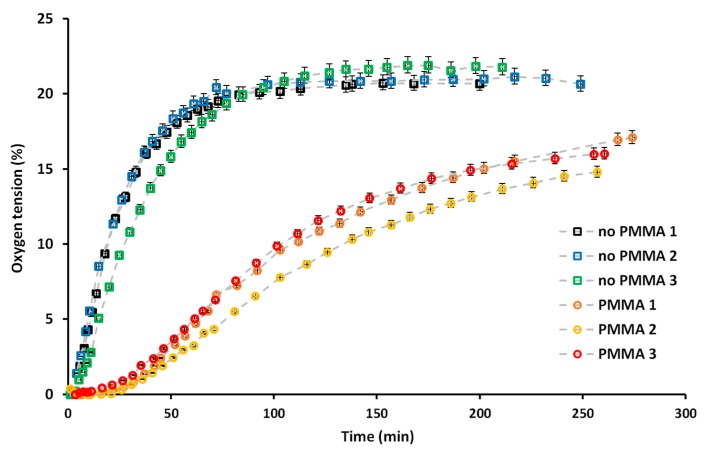
Characterization of the oxygen diffusion in the device over time for devices containing a PMMA sheet (circle symbols) or only PDMS (square symbols), for three independent devices for each case on three different days. Data are presented as the oxygen tension as a function of time, this being assessed by monitoring the decrease in the fluorescence intensity of the tris(2,2′-bipyridyl)dichlororuthenium(II) hexahydrate (Ru(BPY)_3_) oxygen-sensitive probe added in aqueous solution in the device. Data were acquired over a period of more than 4 h, as detailed in the experimental section and analyzed as explained in supplementary material S2.

As a first step, we validated the ability of the PMMA sheet added in the PDMS microfluidic layer to block the oxygen diffusion into the system. To that end, we employed a commercially available Ru-based oxygen-sensitive probe, whose fluorescence is quenched in presence of oxygen. For this first characterization step, a device with a simpler design was employed, which however still exhibited the same essential feature as the tumor-on-a-chip device: a 1-mm wide culture chamber with a height of 150 µm, the same PDMS height above the central chamber and from the same distance between the microfluidic structure and the edge of the device, allowing equivalent diffusion time of the oxygen back to the central chamber compared to the complete design (See Figure S2). In fact, the diffusion rate of oxygen through PMMA (10^−14^ cm^3^ (STP)·cm/cm^2^·s·Pa) [58] is, for the same thickness of material, nine orders of magnitude lower than through PDMS (10^-5^ cm^3^ (STP)·cm/cm^2^·s·Pa) [59]: Therefore, we hypothesize that most of the oxygen diffusion would happen through the lateral sides of the microfluidic device. After both the devices and the oxygen-sensitive probe aqueous solution had been kept overnight in an oxygen-free atmosphere, the solution was pipetted in the devices under the same oxygen-free conditions, to ensure no oxygen was present in the device at the beginning of the characterization. The rate at which oxygen was diffusing back in the culture chamber was measured by monitoring the fluorescence intensity of the oxygen-sensitive probe. It typically decreased upon increase of the oxygen tension to reach a plateau when normoxic conditions (21%) were reached. As depicted on Figure 2 and Figure S3, the presence of the PMMA sheet significantly impacted the rate at which oxygen was diffusing back in the culture chamber. In absence of blocking layer, within ca. 1 h, a plateau in the fluorescence intensity was reached (Figure S3), suggesting atmospheric oxygen tension was achieved in the culture chamber. In sharp contrast, in presence of the PMMA sheet, even after the device had been placed for more than 4 h under atmospheric conditions, the oxygen tension in the culture chamber had not yet reached a plateau, which indicates that the oxygen level was still below atmospheric conditions (Figure 2). Altogether, this characterization step demonstrates the ability of the PMMA layer to block oxygen diffusion from the top of the PDMS device, and that oxygen was solely diffusing through the lateral sides of the device. Therefore, cells grown in the culture chamber should be able to create their own microenvironment and hypoxic conditions upon growth, which we aimed at assessing next.

### 3.3. Cell Culture under Hypoxic Conditions

**Figure 3:**
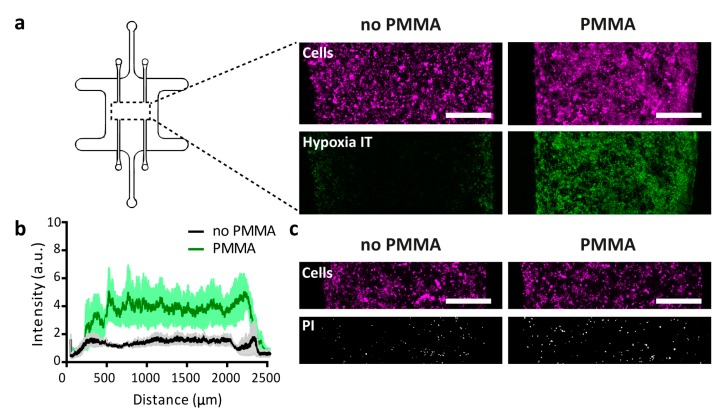
Evaluation of the generation of hypoxia in the microfluidic device. **(a)** 3D tumor cultures of collagen and U-251 MG cells were loaded in devices with or without embedded PMMA sheets, and incubated with the oxygen-sensitive dye ImageIT (green). U-251 MG cells were stained with CytoTell Red (depicted in magenta here). The scale bars represent 500 μm. **(b)** Quantification of the intensity of ImageIT for devices as described in panel **a**. Lines depict averages at distinct positions in the devices and the shaded areas indicate the standard error of the mean (s.e.m.), n = 3. **(c)** Pictures showing similar cell densities of U-251 MG cells (Cytotell Red, magenta) and comparable numbers of PI-positive cells (white) in devices with or without the PMMA sheet. The scale bars represent 500 μm.

The central compartment of microfluidic devices, with or without PMMA sheet, was loaded with a collagen suspension containing 40 million/mL U-251 MG cells. After 48 h of culture in the device, the hypoxia sensor dye Image-IT was added and the oxygen concentration throughout the device characterized. In line with observations from the devices evaluated in absence of cells, a lower oxygen tension was systematically observed in the devices that contained the PMMA sheet as compared to their counterparts without any PMMA sheet (Figure 3a,b). Under the tested conditions, the induced hypoxia did not evidently increase cell death, as similar overall cell density and low amounts of PI-positive cells were found in devices with and without PMMA sheet. In one slice through the entire device, we counted 321 or 515 PI-positive cells in separate devices without PMMA after 48 h of culture, vs. 450 and 577 PI-positive cells in separate devices with the PMMA sheet. In either device, the large majority of cells remained PI negative (Figure 3c,d). Noteworthy, a steep oxygen gradient was found over the first few hundred microns of the 3D culture at either side, with the largest part in the middle of the 2 mm-wide tumor compartment exhibiting similar oxygen levels. These observations are in line with experimental data on oxygen tension drops away from blood vessels measured in vivo in tumors, which showed a very rapid drop in the first 200 μm [60].

Under hypoxic conditions, Ayuso et al. already noticed a significant necrotic core generation after 48 h of growth in a 2 mm-wide polystyrene device for HCT-116 and U-251 MG cells at the same loading density of 40 × 10^6^ million cells/mL in collagen [16]. Since their device was fabricated using an oxygen-impermeable material, their study did not include any control device without hypoxia, so it is unclear to what extent the necrotic cell death they observed was due to hypoxia or to other factors. We did not observe similar reduced viability, nor was there a significant effect of the hypoxia on the cell viability, which is in line with the observation that tumor cells can survive in very low oxygen conditions of around 0.5% [2].

### 3.4. Metabolic Switching of Tumor Cells and Modulation of the Tumor Microenvironment

**Figure 4:**
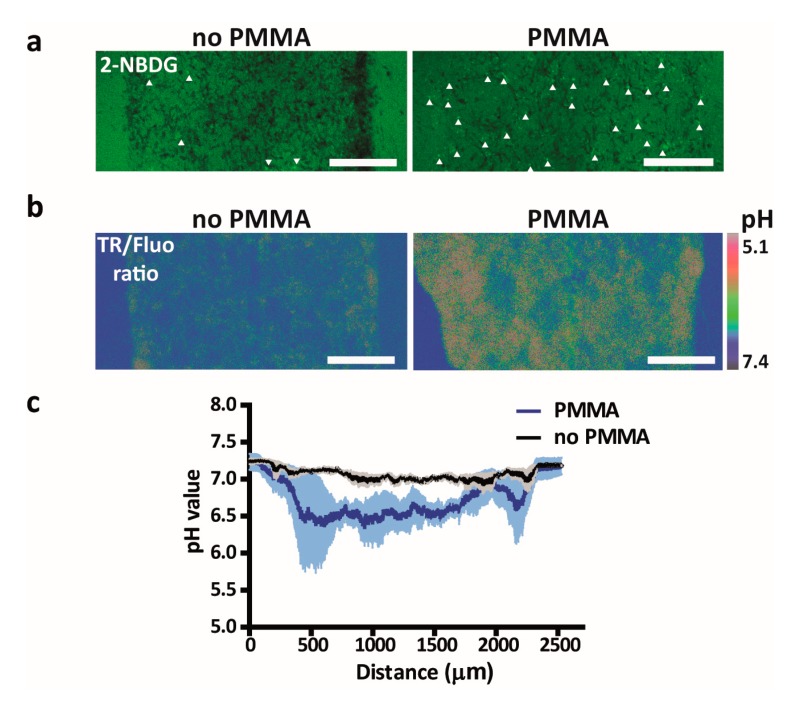
Investigation of the metabolic switch of U-251 MG cells cultured in the tumor-on-a-chip platform. (**a**) 2-NBDG uptake by U-251 MG cells cultured in devices with or without a PMMA sheet. Arrows indicate cells with enhanced 2-NBDG uptake. (**b**) Representative image of the fluorescence ratio of TAMRA and fluorescein (TR/Fluo) of a dual-labeled dextran as a pH sensor in microfluidic devices loaded with U-251 MG cells with or without a PMMA sheet. Scale bars represent 500 μm. (**c**) Quantified pH values in the device based on the ratio of the intensities of the two dyes (TR/Fluo) of the dual-labeled dextran for devices as illustrated in panel b. Lines depict averages at distinct positions in the devices and the shaded areas indicate the s.e.m.

To investigate whether tumor cells were metabolically affected by the lower oxygen tension, we incubated U-251 MG cells after 2 days of culture in the microfluidic devices with the glucose analog 2-NBDG, of which a higher uptake reflects increased glucose consumption. Up to this point, we expect no significant glucose depletion given the large excess of glucose in the medium (12 μmol glucose considering a total volume of medium of 500 μL [with 25 mM glucose], refreshed every 24 h) and the low expected consumption of the < 10,000 cells in the device (assuming a maximum glucose consumption rate of 800 fmol per cell per hour [61], the glucose consumption will be less than 200 nmol/24 h). Through a 90-min time lapse of 2-NDBG diffusion and uptake, we indeed noticed a significant increase in 2-NBDG uptake by cells grown in the device with the PMMA sheet as compared to cells grown in control devices without the PMMA sheet, reflecting a switch towards a more glycolytic behavior (Figure 4a). A similar switch in metabolism has been reported for a number of microfluidic devices, albeit in cancer cells grown in 2D rather than 3D conditions. For instance, Rexius-Hall et al. reported that hypoxia in their device induced up-regulation of the glycolysis-associated proteins glucose transporter 1 (Glut-1), LDHA, PDK1-3 as well as nuclear translocation of the subunit of HIF1α, which together with HIF1β forms HIF1, a key hypoxia-responsive transcription factor [62]. Similarly, Ando et al. observed upregulation of Glut-1 in addition to several other glycolysis markers under hypoxic conditions [63]. Finally, Ayuso et al. described differences in 2-NBDG diffusion rates in 3D tumor cell cultures grown under hypoxic conditions, potentially reflecting differences in glycolytic behavior between cells, though the effect of hypoxia itself on metabolic behavior was not investigated [16].

Since a greater reliance on glycolysis for energy production causes a buildup of protons, a decreased pH in the tumor microenvironment usually ensues, which is widely seen in vivo and positively correlates with hypoxia [60,64]. To investigate whether the pH in the tumor microenvironment would be influenced, a pH-sensing dual-labeled dextran was employed. As expected, a significant decrease in the tumor microenvironmental pH was found in the 3D culture grown in the device with the PMMA sheet as compared to the one without the PMMA sheet (Figure 4b,c). To the best of our knowledge, this is the first report of a microfluidic device containing a 3D tumor cell culture that directly shows an effect on pH levels, while this parameter is essential for recreating the in vivo tumor microenvironment, especially when investigating novel (immuno)therapies. Notably, mild or moderate hypoxia by itself does not hamper T cell function, but an acidic environment potently suppresses the cytotoxic activity of T cells, which play an essential role in antibody-based immunotherapies [9].

## 4. Conclusions

We report here a tumor-on-a-chip platform that can easily be fabricated without having access to a cleanroom environment, and in which normoxic or hypoxic conditions can reproducibly be generated in a 3D collagen-based tumor model, while being operated in a standard incubator. A first-stage characterization of the microfluidic devices revealed efficient blocking of oxygen diffusion by the PMMA sheet embedded in the PDMS fluidic layer as well as the presence of hypoxic conditions in the 3D tumor cell culture. Under such hypoxic conditions, cells in the microfluidic devices were found to switch their metabolism towards a more glycolytic behavior and to acidify the on-chip tumor environment. Altogether, our tumor-on-a-chip platform is suitable for recreating key aspects of the tumor environment, which will be instrumental in studying in vitro metabolic changes or tumor therapeutic responses affected by the hypoxic tumor microenvironment.

## Supplementary Materials

The following are available online at https://www.mdpi.com/2072-666X/11/4/382/s1, Supplementary material Figure S1: Schematic representation of the entire fabrication process of the tumor-on-a-chip platform, illustrating how the position of the PMMA sheet in the PDMS layer is controlled and how device bonding is operated with the cotton threads;. Supplementary material Figure S2: Detailed designs of the molds employed to fabricate the two devices (culture devices and devices for hypoxia characterization): SolidWorks sketches and corresponding pictures of these molds; Supplementary material Figure S3: Details on the data analysis for the oxygen diffusion characterization in the microfluidic devices.
