# Supplementary material for: Metabolic Switching of Tumor Cells under Hypoxic Conditions in a Tumor-on-a-chip Model
Source: Micromachines (Basel). 2020 Apr 4;11(4):382. doi: 10.3390/mi11040382 (PMC7231186; doi:10.3390/mi11040382)
Supplement: Supplementary file 1 [file micromachines-11-00382-s001.pdf]

micromachines

Supplementary Materials

Metabolic Switching of Tumor Cells under Hypoxic Conditions in a Tumor-on-a-chip Model

Valentina Palacio-Castañeda [1], Lucas Kooijman [2,†], Bastien Venzac [2,†], Wouter P.R. Verdurmen [1,*] and Séverine Le Gac [2,*]

1 Department of Biochemistry, Radboud Institute for Molecular Life Sciences (RIMLS), Radboud University Medical Center, Geert Grooteplein 28, 6525 GA Nijmegen, The Netherlands; Valentina.Palacio-Castaneda@radboudumc.nl

2 Applied Microfluidics for BioEngineering Research, TechMed Center & MESA+ Institute for Nanotechnology, University of Twente, Postbus 217, 7500AE Enschede, The Netherlands; l.j.kooijman@utwente.nl (L.K.); b.venzac@utwente.nl (B.V.)

* Correspondence: S.LeGac@utwente.nl (S.L.G.); Wouter.Verdurmen@radboudumc.nl (W.P.R.V.)

† These authors have equally contributed to this work

Supplementary information 1—Schematic representation of the entire fabrication process of the tumor-on-a-chip platform

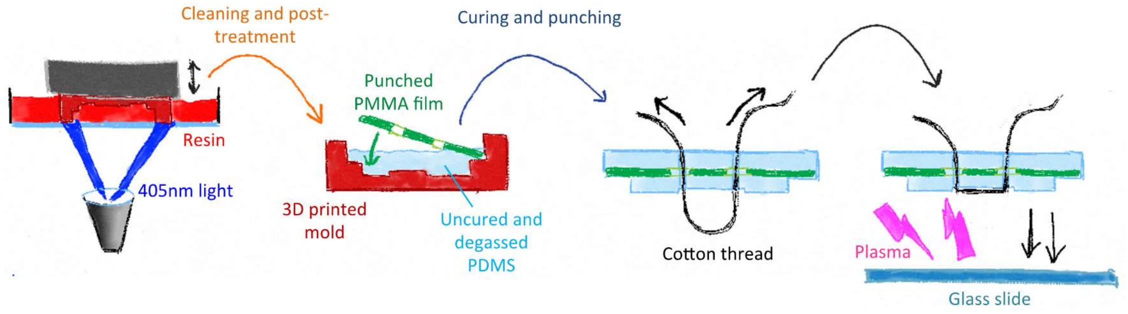

Figure S1: Schematic representation of the entire fabrication process of the tumor-on-a-chip platform. First, a mold is 3D-printed using stereolithography (SLA). After a cleaning and post-treatment step, the mold is filled with uncured PDMS (10:1 prepolymer:curing agent weight ratio), and a thin PMMA sheet, in which holes were already machined, is carefully introduced on dedicated structures embedded in the 3D printed mold (See Figure S2). After curing, reservoirs are punched into the PDMS and two pieces of cotton threads are introduced in two guiding channels lining the culture chamber. After plasma treatment of both a microscope glass slide and the PDMS microfluidic layer, device assembly is performed. For control microsystems without any PMMA sheet, the fabrication process is the same expect the PMMA sheet introduction step, which is omitted.

Supplementary information 2—Detailed designs of the microfluidic devices

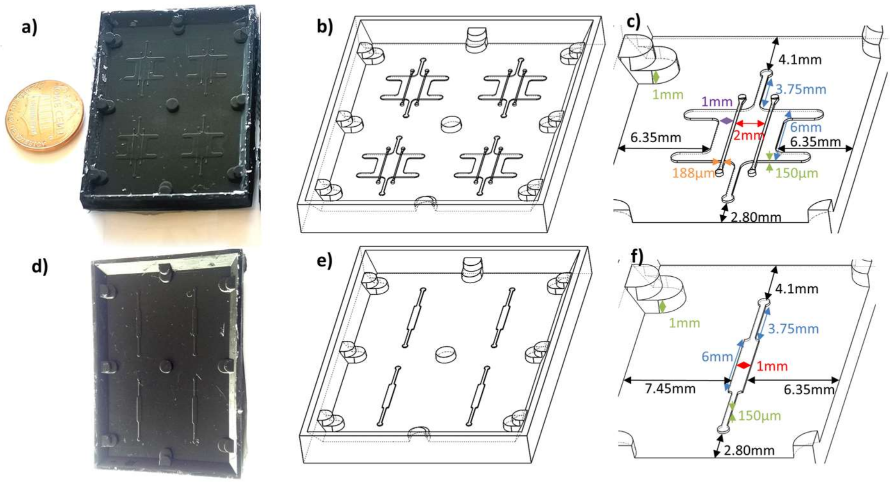

Figure 2. SolidWorks designs and corresponding pictures of the different molds employed to fabricate the two devices: (**a**–**c**) For the cell culture experiments and (**d**–**f**) the oxygen diffusion characterization. (**a**) Photograph of the 3D-printed mold used for the soft-lithography production of the tumor-on-a-chip device; (**b**) SolidWorks sketch of the same structures; and (**c**) enlarged view of one device showing all relevant dimensions. (**d**) Photograph of the 3D printed mold used to produce devices with the simplified design for the oxygen diffusion evaluation; (**e**) Solidworks sketch of the same structure; and (**f**) enlarged view on one device showing all relevant dimensions.

Supplementary information 3—Data analysis for the characterization of the microfluidic systems under normoxic and hypoxic conditions

We measured the fluorescence intensity of the oxygen-sensitive probe as a function of time in devices equipped or not with a PMMA sheet. These data were first plotted as relative fluorescence intensity values, using the fluorescence intensity value measured at the beginning of the experiments: this value was assumed to represent 0% oxygen and corresponded to the highest fluorescence intensity measured. As shown below (Figure S2), in absence of PMMA sheet, after typically one hour, a plateau is reached indicated that the oxygen tension has reached atmospheric conditions (21%).

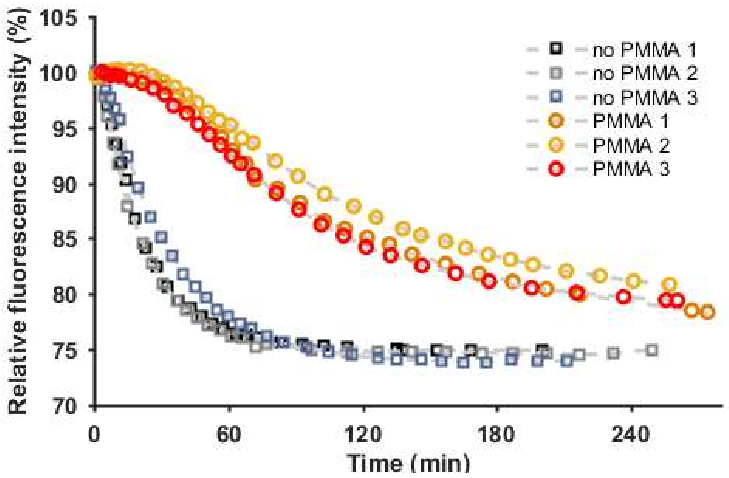

Figure S3. Characterization of the oxygen diffusion in the device over time for devices containing a PMMA sheet (circle symbols) or only PDMS (square symbols), for three independent devices for each case on three different days. Data are presented as the decrease in the fluorescence intensity of the tris(2,2′-bipyridyl)dichlororuthenium(II) hexahydrate ($Ru(BPY)_3$) oxygen-sensitive probe added in aqueous solution in the device. Data were acquired over a period of more than 4 hours, as detailed in the experimental section. Data are normalized to the fluorescence intensity measured at the start of the experiment, under oxygen-free conditions.

As a next step, we determined the oxygen tension variations in the device as a function of time. The relationship between the measured fluorescence and the oxygen concentration is described by the Stern-Volmer equation: $\frac{I_0}{I} = 1 + K_q[O_2]$.

Where I_0 is the fluorescence intensity in absence of oxygen (0% O_2); I the fluorescence intensity measured at a given oxygen tension; and K_q the quenching constant.

We first determined K_q using data extracted from the three experiments using microfluidic systems without the PMMA film, as $K_q = \frac{I_0 - I_{21}}{I_{21} * 21}$. As mentioned above, for each experiment, I_0 is the maximum value measured and we averaged the value of I during the plateau (after *ca.* 100 min) to obtain $I_{21\%}$.

Experiment	I_0 (a.u.)	$I_{21\%}$ (a.u.)	K_q
No PMMA 1	600.7	450.9	0.0158
No PMMA 2	615.1	459.4	0.0161
No PMMA 3	665.8	492.6	0.0166

This yields the average value of K_q=0.0162 ± 0.0004

The oxygen tension at each time point was next calculated using the following equation:

$$[O_2] = \frac{\frac{I_0}{I} - 1}{K_q}$$

Error bars for the data presented in Figure 2 in the main paper were obtained for every measurement point using the extreme values of K_q.
